# Draft genome sequence of *Bacillus anthracis* strains, isolated from soil samples from a historic tannery site in Upper Austria

**DOI:** 10.1128/mra.00932-24

**Published:** 2025-03-05

**Authors:** Maximilian F. Mayerhofer-Rochel, Florian Himmelbauer, Hans-Jörg Hellinger, Michael P. Szostak, Gregor Grass, Monika Ehling-Schulz

**Affiliations:** 1 Department of Pathobiology, Functional Microbiology, Institute of Microbiology, University of Veterinary Medicine Vienna 27260 , Vienna, Austria; 2 Armaments and Defence Technology Agency, NBC & Environmental Protection Technology Division, Vienna, Austria; 3 Bundeswehr Institute of Microbiology (IMB) 539152 , Munich, Germany; The University of ArizonaPlant Sciences, Tucson, Arizona, USA

**Keywords:** anthrax, soil, tannery, Austria

## Abstract

In this announcement, we present the draft genomes of four *Bacillus anthracis* isolates, MH-MFM, MH-VW, MH-PR, and MH-JJ, originating from soil samples retrieved from a sludge disposal site of a historic tannery site in Upper Austria.

## ANNOUNCEMENT

*Bacillus anthracis*, a gram-positive, spore-forming bacterium of the *Bacillus cereus* group, causes the zoonotic disease anthrax and can remain viable in soil for decades. Such sites threaten veterinarian and public health. As the last outbreak of anthrax in Austria occurred at a pasture site in Tyrol in 1988, historic anthrax loci may be a concern for prospective disease monitoring. Soil samples were taken using a hand auger at an abandoned tannery in Upper Austria and were screened for *B. anthracis* by cultivation on semi-selective agar plates (1). Single colonies of isolates, classified as *B. anthracis* after confirmation of the presence of *B. anthracis*-specific markers by real-time PCR assays (2, 3), were stored using Microbank (Pro-Lab Diagnostics Inc., Canada). Canonical single-nucleotide polymorphism-based genotyping revealed that all four isolates belong to A.Br.064, previously not yet associated with Austria (1).

For whole genome sequencing (WGS) analysis, *B. anthracis* isolates were retrieved from Microbank and grown on StdI agar (Merck KGaA, Germany) at 37°C. DNA from colonies was isolated using the MasterPure Total DNA/RNA Purification kit (Lucigen Middleton, USA) with a pre-lysis step using 24,839 U of Ready-to-Lyse lysozyme solution (Lucigen Middleton, USA) for 60 minutes at 37°C.

Illumina paired-end sequencing was performed using ILMN DNA LP (M) tagmentation (24 Samples, IPB) for library preparation and the MiniSeq Mid Output Kit (300 cycles) on the MiSeq system (Illumina, USA). The run produced 4.64 GB with an average Q30 of 91.57 GB. Nanopore single-end sequencing was performed from the same DNA extract using SQK-LSK109 chemistry on an R10.4.1 flow cell on the MinION system (Oxford Nanopore Technologies, UK), running system software MinKNOW 23.07.8 for the generation of long reads for hybrid assembly. The library (run time: 50 h) resulted in 1.19 million reads, with estimated 2.83 Gb and 6.51 kb N50. Base calling was performed using fast mode with 400 bps settings.

MinION raw reads were assembled using FLYE v.2.9.2-b1786 (4). Illumina raw reads were mapped against the assembled FLYE output using BWA v.0.7.17-r1188 (5) resulting in a hybrid assembly for chromosome, pXO1, and pXO2, respectively. The raw hybrid assembly was polished using Pilon v.1.24 (6). Ragtag v.02.01.00 (7) was used for reference-guided assembly to polish assemblies and reduce the number of contigs (see Table 1) to three (according to chromosome, plasmids pXO1 and pXO2). Default parameters were used for all software.

**TABLE 1:** Summary of sequencing results

Strain biosample	Chromosome coverage (MinION/Illumina)	Plasmid pXO1 coverage (MinION/Illumina)	Plasmid pXO2 coverage (MinION/Illumina)	Number of contigs pre-polishing	N50 of pre-polished contigs	Length (bp) of chromosome pXO1 and pXO2	CheckM BUSCO	GC%	Number of CDS	SRA assembly
MH-MFM SAMN38634750	91×/21×	347×/58×	228×/33×	13	3905291	5,175,977 181,707 94,821	98.85 98.9	35.1	5,620	SRX23276499 SRX23276495 CP140729 CP140730 CP140731
MH-VW SAMN38634753	41×/37×	117×/88×	75×/57×	5	5228373	5,228,373 181,736 94,805	99.43 99.3	35.2	5,625	SRX23276496 SRX23276492 CP140720 CP140721 CP140722
MH-PR SAMN38634751	38×/58×	133×/154×	81×/94×	5	5228464	5,228,464 181,719 94,815	99.43 99.1	35.2	5,627	SRX23276498 SRX23276494 CP140726 CP140727 CP140728
MH-JJ SAMN38634752	78×/19×	328×/50×	179×/32×	8	5227439	5,227,439 180,368 94,722	99.43 99.1	35.2	5,639	SRX23276497 SRX23276493 CP140723 CP140724 CP140725

The polished replicons, corresponding to the chromosome and plasmids pXO1 and pXO2, were subjected to CheckM v.01.02.02 (8), BUSCO v.05.02.2002 (9) with baciallales marker, Geneious Prime v.2023.1.2, and PGAP V2023-10-03.build7061 (10) for a check of completeness, contamination, GC content, and protein coding sequence estimate (Table 1). The 5.2 MB chromosomes were submitted to the pubMLST database (11) to confirm the species classification of *B. anthracis*.

## Data Availability

Raw reads and assembly data of chromosomes, pXO1 and pXO2 have been deposited at NCBI Bioproject PRJNA1048328. The version described in this paper is the first version.
